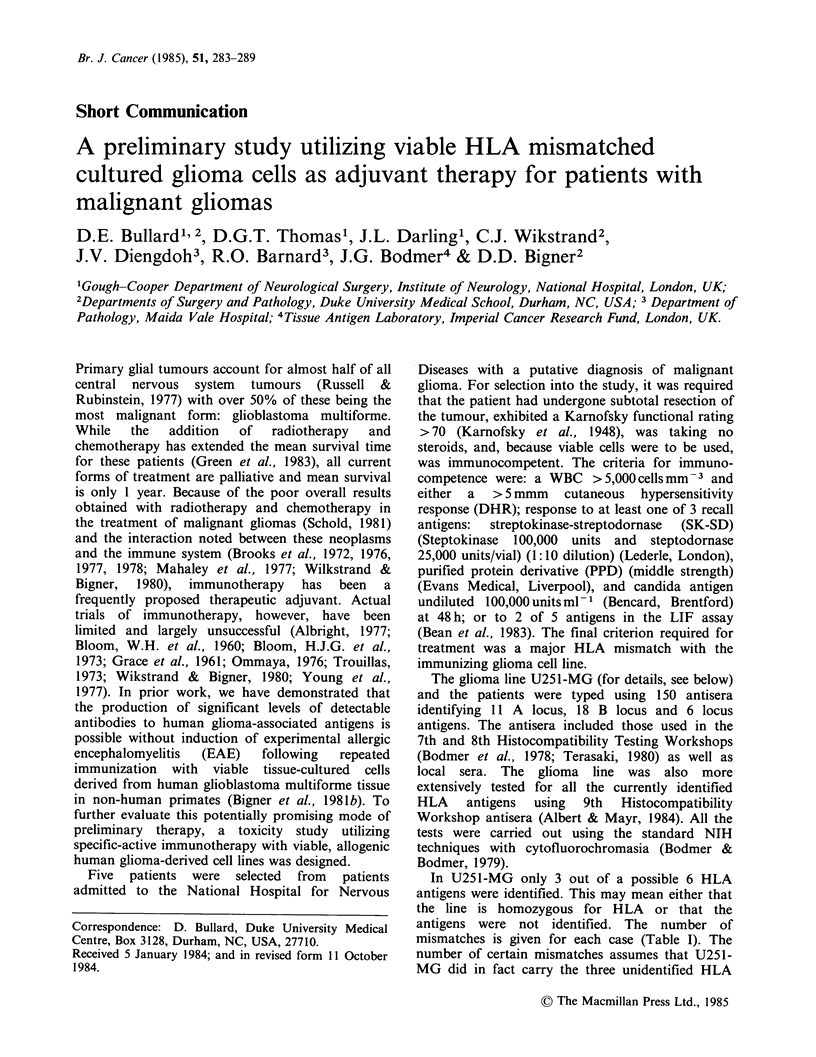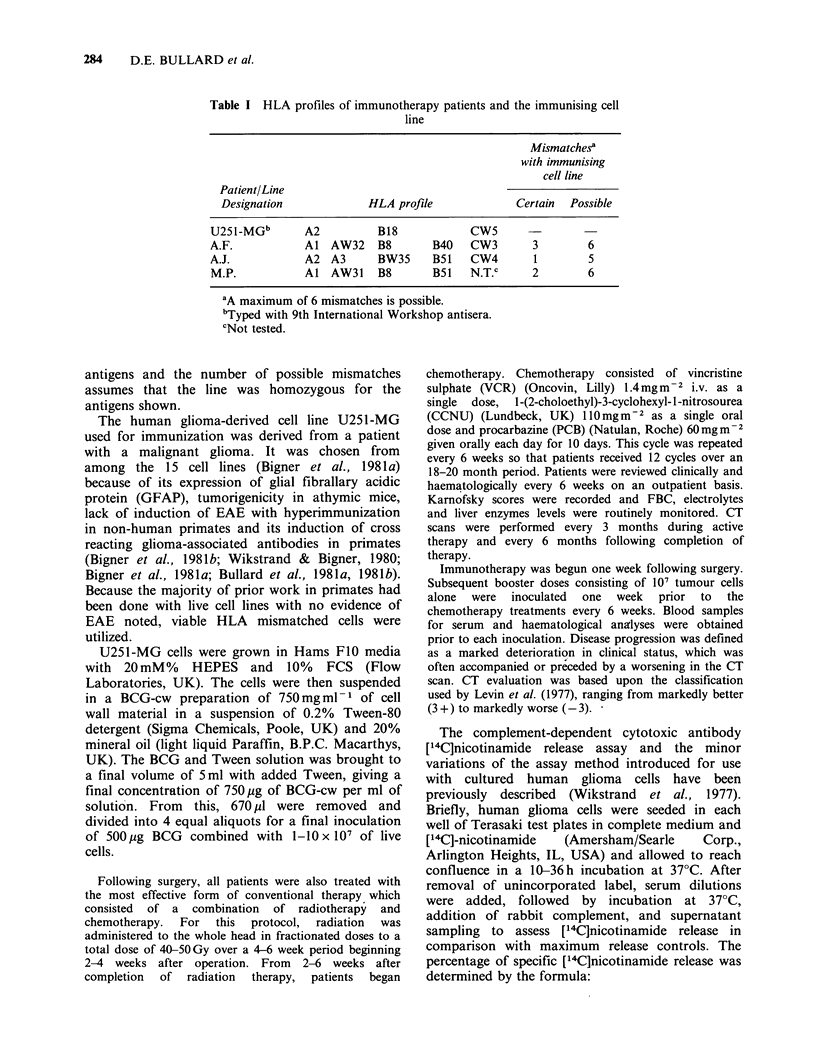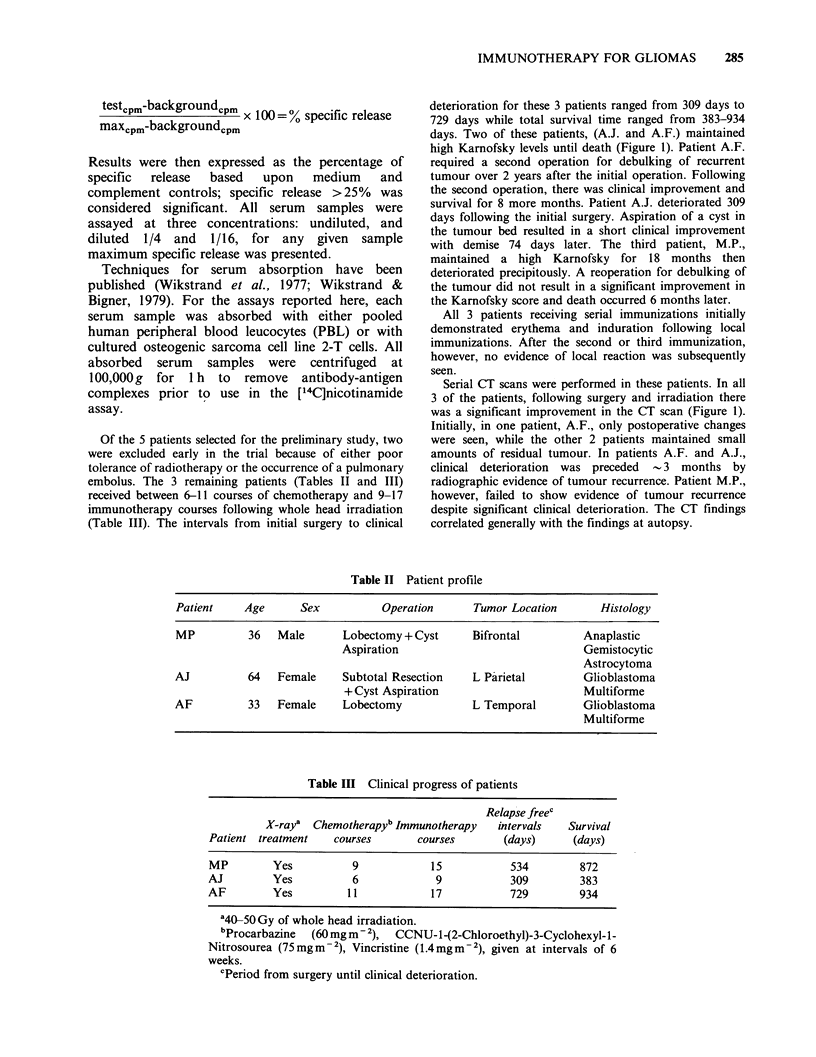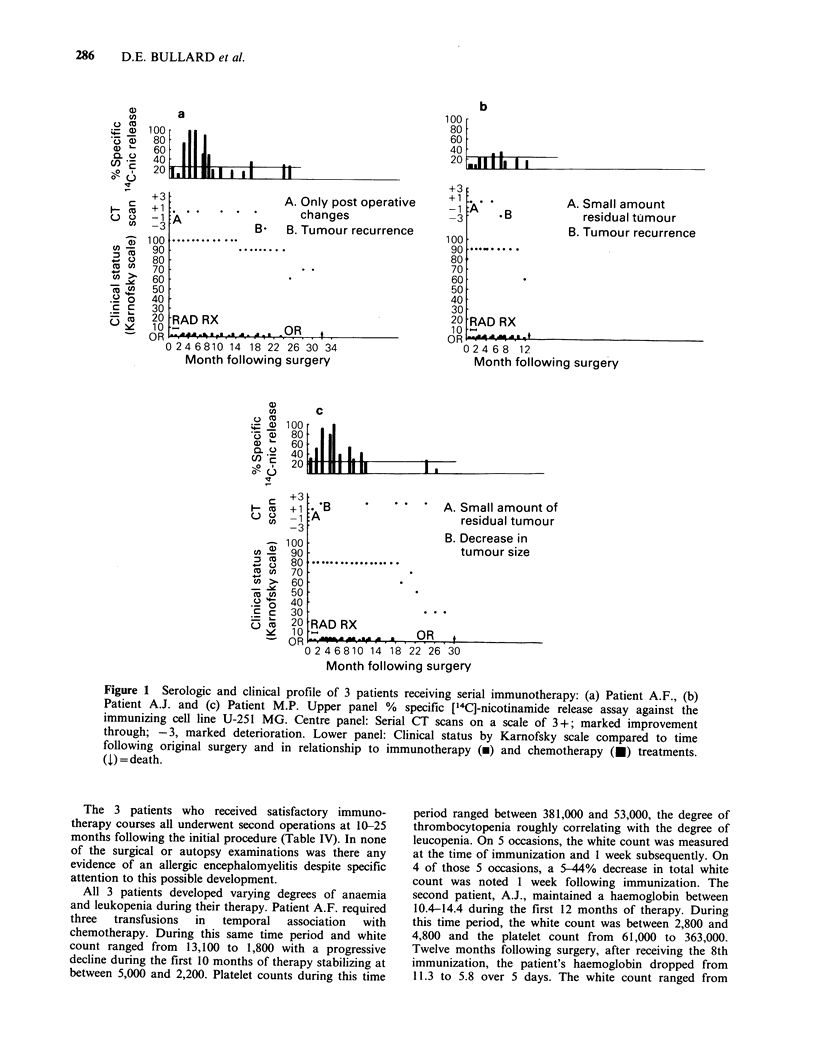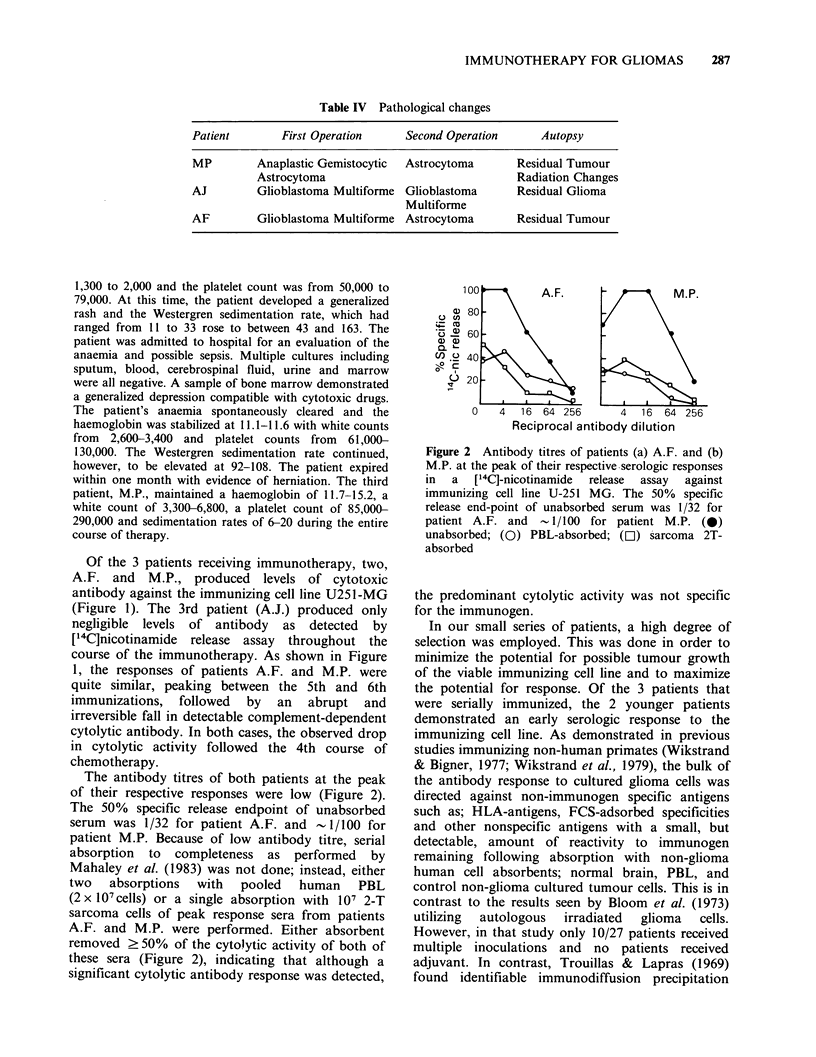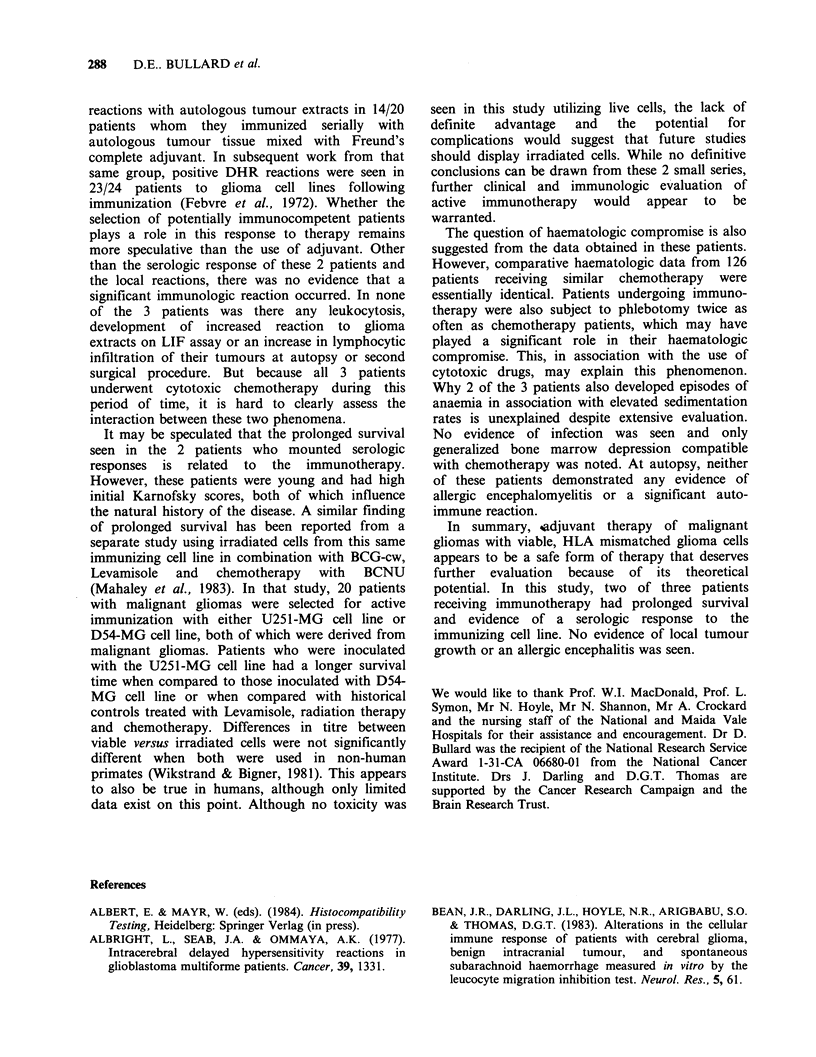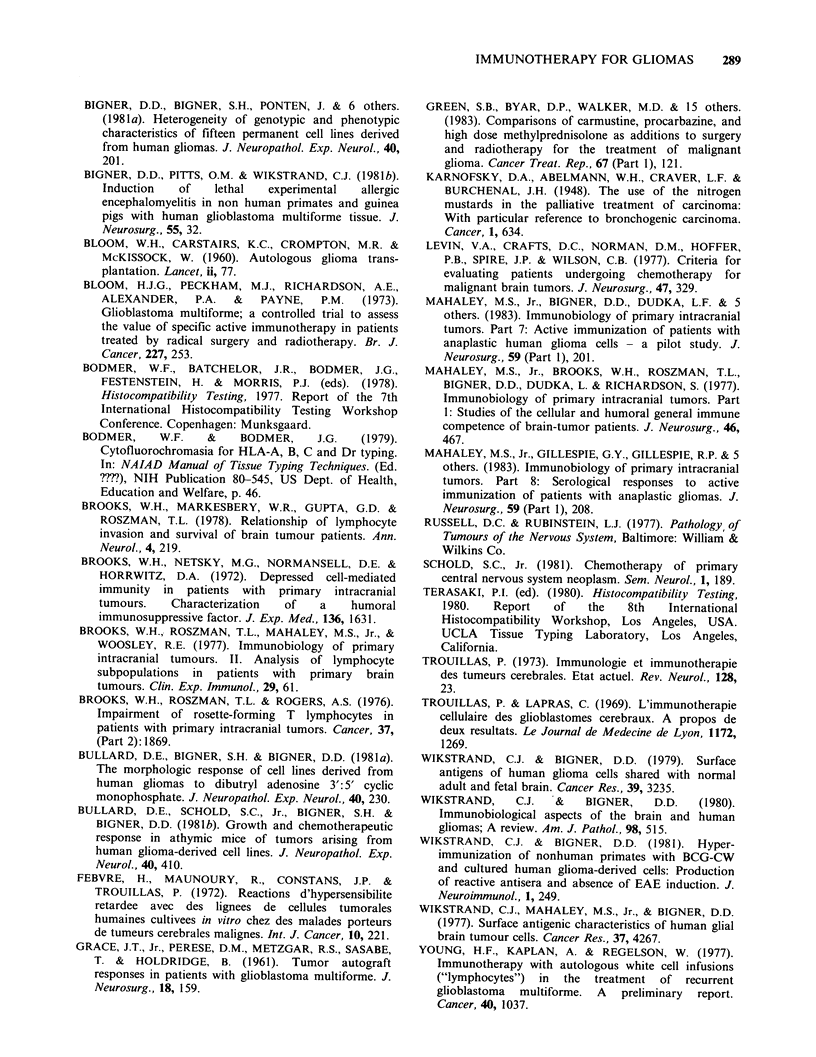# A preliminary study utilizing viable HLA mismatched cultured glioma cells as adjuvant therapy for patients with malignant gliomas.

**DOI:** 10.1038/bjc.1985.41

**Published:** 1985-02

**Authors:** D. E. Bullard, D. G. Thomas, J. L. Darling, C. J. Wikstrand, J. V. Diengdoh, R. O. Barnard, J. G. Bodmer, D. D. Bigner


*Br. J. Cancer* (1985), **51**, 283–289

**Short Communication**

### A preliminary study utilizing viable HLA mismatched cultured glioma cells as adjuvant therapy for patients with malignant gliomas

D.E. Bullard[1,2], D.G.T. Thomas[1], J.L. Darling[1], C.J. Wikstrand[2], J.V. Diengdoh[3], R.O. Barnard[3], J.G. Bodmer[4] & D.D. Bigner[2]

[1]*Gough–Cooper Department of Neurological Surgery, Institute of Neurology, National Hospital, London, UK;* [2]*Departments of Surgery and Pathology, Duke University Medical School, Durham, NC, USA;* [3] *Department of Pathology, Maida Vale Hospital;* [4]*Tissue Antigen Laboratory, Imperial Cancer Research Fund, London, UK.*

Primary glial tumours account for almost half of all central nervous system tumours (Russell & Rubinstein, 1977) with over 50% of these being the most malignant form: glioblastoma multiforme. While the addition of radiotherapy and chemotherapy has extended the mean survival time for these patients (Green *et al.*, 1983), all current forms of treatment are palliative and mean survival is only 1 year. Because of the poor overall results obtained with radiotherapy and chemotherapy in the treatment of malignant gliomas (Schold, 1981) and the interaction noted between these neoplasms and the immune system (Brooks *et al.*, 1972, 1976, 1977, 1978; Mahaley *et al.*, 1977; Wilkstrand & Bigner, 1980), immunotherapy has been a frequently proposed therapeutic adjuvant. Actual trials of immunotherapy, however, have been limited and largely unsuccessful (Albright, 1977; Bloom, W.H. *et al.*, 1960; Bloom, H.J.G. *et al.*, 1973; Grace *et al.*, 1961; Ommaya, 1976; Trouillas, 1973; Wikstrand & Bigner, 1980; Young *et al.*, 1977). In prior work, we have demonstrated that the production of significant levels of detectable antibodies to human glioma-associated antigens is possible without induction of experimental allergic encephalomyelitis (EAE) following repeated immunization with viable tissue-cultured cells derived from human glioblastoma multiforme tissue in non-human primates (Bigner *et al.*, 1981*b*). To further evaluate this potentially promising mode of preliminary therapy, a toxicity study utilizing specific-active immunotherapy with viable, allogenic human glioma-derived cell lines was designed.

Five patients were selected from patients admitted to the National Hospital for Nervous Diseases with a putative diagnosis of malignant glioma. For selection into the study, it was required that the patient had undergone subtotal resection of the tumour, exhibited a Karnofsky functional rating >70 (Karnofsky *et al.*, 1948), was taking no steroids, and, because viable cells were to be used, was immunocompetent. The criteria for immunocompetence were: a WBC $>5{,}000\,\text{cells}\,\text{mm}^{-3}$ and either a >5 mmm cutaneous hypersensitivity response (DHR); response to at least one of 3 recall antigens: streptokinase-streptodornase (SK-SD) (Steptokinase 100,000 units and steptodornase 25,000 units/vial) (1:10 dilution) (Lederle, London), purified protein derivative (PPD) (middle strength) (Evans Medical, Liverpool), and candida antigen undiluted $100{,}000\,\text{units}\,\text{ml}^{-1}$ (Bencard, Brentford) at 48 h; or to 2 of 5 antigens in the LIF assay (Bean *et al.*, 1983). The final criterion required for treatment was a major HLA mismatch with the immunizing glioma cell line.

The glioma line U251-MG (for details, see below) and the patients were typed using 150 antisera identifying 11 A locus, 18 B locus and 6 locus antigens. The antisera included those used in the 7th and 8th Histocompatibility Testing Workshops (Bodmer *et al.*, 1978; Terasaki, 1980) as well as local sera. The glioma line was also more extensively tested for all the currently identified HLA antigens using 9th Histocompatibility Workshop antisera (Albert & Mayr, 1984). All the tests were carried out using the standard NIH techniques with cytofluorochromasia (Bodmer & Bodmer, 1979).

In U251-MG only 3 out of a possible 6 HLA antigens were identified. This may mean either that the line is homozygous for HLA or that the antigens were not identified. The number of mismatches is given for each case (Table I). The number of certain mismatches assumes that U251-MG did in fact carry the three unidentified HLA

Correspondence: D. Bullard, Duke University Medical Centre, Box 3128, Durham, NC, USA, 27710.
Received 5 January 1984; and in revised form 11 October 1984.

© The Macmillan Press Ltd., 1985

**Table I** HLA profiles of immunotherapy patients and the immunising cell line

| *Patient/Line Designation* | *HLA profile* | | | | | *Mismatches[a] with immunising cell line* | |
|---|---|---|---|---|---|---|---|
| | | | | | | *Certain* | *Possible* |
| U251-MG[b] | A2 | | B18 | | CW5 | — | — |
| A.F. | A1 | AW32 | B8 | B40 | CW3 | 3 | 6 |
| A.J. | A2 | A3 | BW35 | B51 | CW4 | 1 | 5 |
| M.P. | A1 | AW31 | B8 | B51 | N.T.[c] | 2 | 6 |

[a]A maximum of 6 mismatches is possible.
[b]Typed with 9th International Workshop antisera.
[c]Not tested.

antigens and the number of possible mismatches assumes that the line was homozygous for the antigens shown.

The human glioma-derived cell line U251-MG used for immunization was derived from a patient with a malignant glioma. It was chosen from among the 15 cell lines (Bigner *et al.*, 1981*a*) because of its expression of glial fibrallary acidic protein (GFAP), tumorigenicity in athymic mice, lack of induction of EAE with hyperimmunization in non-human primates and its induction of cross reacting glioma-associated antibodies in primates (Bigner *et al.*, 1981*b*; Wikstrand & Bigner, 1980; Bigner *et al.*, 1981*a*; Bullard *et al.*, 1981*a*, 1981*b*). Because the majority of prior work in primates had been done with live cell lines with no evidence of EAE noted, viable HLA mismatched cells were utilized.

U251-MG cells were grown in Hams F10 media with 20 mM% HEPES and 10% FCS (Flow Laboratories, UK). The cells were then suspended in a BCG-cw preparation of 750 mg ml$^{-1}$ of cell wall material in a suspension of 0.2% Tween-80 detergent (Sigma Chemicals, Poole, UK) and 20% mineral oil (light liquid Paraffin, B.P.C. Macarthys, UK). The BCG and Tween solution was brought to a final volume of 5 ml with added Tween, giving a final concentration of 750 $\mu$g of BCG-cw per ml of solution. From this, 670 $\mu$l were removed and divided into 4 equal aliquots for a final inoculation of 500 $\mu$g BCG combined with 1–10 $\times 10^7$ of live cells.

Following surgery, all patients were also treated with the most effective form of conventional therapy which consisted of a combination of radiotherapy and chemotherapy. For this protocol, radiation was administered to the whole head in fractionated doses to a total dose of 40–50 Gy over a 4–6 week period beginning 2–4 weeks after operation. From 2–6 weeks after completion of radiation therapy, patients began chemotherapy. Chemotherapy consisted of vincristine sulphate (VCR) (Oncovin, Lilly) 1.4 mg m$^{-2}$ i.v. as a single dose, 1-(2-choloethyl)-3-cyclohexyl-1-nitrosourea (CCNU) (Lundbeck, UK) 110 mg m$^{-2}$ as a single oral dose and procarbazine (PCB) (Natulan, Roche) 60 mg m$^{-2}$ given orally each day for 10 days. This cycle was repeated every 6 weeks so that patients received 12 cycles over an 18–20 month period. Patients were reviewed clinically and haematologically every 6 weeks on an outpatient basis. Karnofsky scores were recorded and FBC, electrolytes and liver enzymes levels were routinely monitored. CT scans were performed every 3 months during active therapy and every 6 months following completion of therapy.

Immunotherapy was begun one week following surgery. Subsequent booster doses consisting of $10^7$ tumour cells alone were inoculated one week prior to the chemotherapy treatments every 6 weeks. Blood samples for serum and haematological analyses were obtained prior to each inoculation. Disease progression was defined as a marked deterioration in clinical status, which was often accompanied or preceded by a worsening in the CT scan. CT evaluation was based upon the classification used by Levin *et al.* (1977), ranging from markedly better (3+) to markedly worse (−3).

The complement-dependent cytotoxic antibody [$^{14}$C]nicotinamide release assay and the minor variations of the assay method introduced for use with cultured human glioma cells have been previously described (Wikstrand *et al.*, 1977). Briefly, human glioma cells were seeded in each well of Terasaki test plates in complete medium and [$^{14}$C]-nicotinamide (Amersham/Searle Corp., Arlington Heights, IL, USA) and allowed to reach confluence in a 10–36 h incubation at 37°C. After removal of unincorporated label, serum dilutions were added, followed by incubation at 37°C, addition of rabbit complement, and supernatant sampling to assess [$^{14}$C]nicotinamide release in comparison with maximum release controls. The percentage of specific [$^{14}$C]nicotinamide release was determined by the formula:

$$\frac{\text{test}_{cpm}\text{-background}_{cpm}}{\text{max}_{cpm}\text{-background}_{cpm}} \times 100 = \% \text{ specific release}$$

Results were then expressed as the percentage of specific release based upon medium and complement controls; specific release >25% was considered significant. All serum samples were assayed at three concentrations: undiluted, and diluted 1/4 and 1/16, for any given sample maximum specific release was presented.

Techniques for serum absorption have been published (Wikstrand *et al.,* 1977; Wikstrand & Bigner, 1979). For the assays reported here, each serum sample was absorbed with either pooled human peripheral blood leucocytes (PBL) or with cultured osteogenic sarcoma cell line 2-T cells. All absorbed serum samples were centrifuged at 100,000 *g* for 1 h to remove antibody-antigen complexes prior to use in the [$^{14}$C]nicotinamide assay.

Of the 5 patients selected for the preliminary study, two were excluded early in the trial because of either poor tolerance of radiotherapy or the occurrence of a pulmonary embolus. The 3 remaining patients (Tables II and III) received between 6–11 courses of chemotherapy and 9–17 immunotherapy courses following whole head irradiation (Table III). The intervals from initial surgery to clinical deterioration for these 3 patients ranged from 309 days to 729 days while total survival time ranged from 383–934 days. Two of these patients, (A.J. and A.F.) maintained high Karnofsky levels until death (Figure 1). Patient A.F. required a second operation for debulking of recurrent tumour over 2 years after the initial operation. Following the second operation, there was clinical improvement and survival for 8 more months. Patient A.J. deteriorated 309 days following the initial surgery. Aspiration of a cyst in the tumour bed resulted in a short clinical improvement with demise 74 days later. The third patient, M.P., maintained a high Karnofsky for 18 months then deteriorated precipitously. A reoperation for debulking of the tumour did not result in a significant improvement in the Karnofsky score and death occurred 6 months later.

All 3 patients receiving serial immunizations initially demonstrated erythema and induration following local immunizations. After the second or third immunization, however, no evidence of local reaction was subsequently seen.

Serial CT scans were performed in these patients. In all 3 of the patients, following surgery and irradiation there was a significant improvement in the CT scan (Figure 1). Initially, in one patient, A.F., only postoperative changes were seen, while the other 2 patients maintained small amounts of residual tumour. In patients A.F. and A.J., clinical deterioration was preceded ~3 months by radiographic evidence of tumour recurrence. Patient M.P., however, failed to show evidence of tumour recurrence despite significant clinical deterioration. The CT findings correlated generally with the findings at autopsy.

**Table II** Patient profile

| *Patient* | *Age* | *Sex* | *Operation* | *Tumor Location* | *Histology* |
|---|---|---|---|---|---|
| MP | 36 | Male | Lobectomy + Cyst Aspiration | Bifrontal | Anaplastic Gemistocytic Astrocytoma |
| AJ | 64 | Female | Subtotal Resection + Cyst Aspiration | L Parietal | Glioblastoma Multiforme |
| AF | 33 | Female | Lobectomy | L Temporal | Glioblastoma Multiforme |

**Table III** Clinical progress of patients

| *Patient* | *X-ray*[a] *treatment* | *Chemotherapy*[b] *courses* | *Immunotherapy courses* | *Relapse free*[c] *intervals (days)* | *Survival (days)* |
|---|---|---|---|---|---|
| MP | Yes | 9 | 15 | 534 | 872 |
| AJ | Yes | 6 | 9 | 309 | 383 |
| AF | Yes | 11 | 17 | 729 | 934 |

[a]40–50 Gy of whole head irradiation.

[b]Procarbazine (60 mg m$^{-2}$), CCNU-1-(2-Chloroethyl)-3-Cyclohexyl-1-Nitrosourea (75 mg m$^{-2}$), Vincristine (1.4 mg m$^{-2}$), given at intervals of 6 weeks.

[c]Period from surgery until clinical deterioration.

**Figure 1** Serologic and clinical profile of 3 patients receiving serial immunotherapy: (a) Patient A.F., (b) Patient A.J. and (c) Patient M.P. Upper panel % specific [$^{14}$C]-nicotinamide release assay against the immunizing cell line U-251 MG. Centre panel: Serial CT scans on a scale of 3+; marked improvement through; −3, marked deterioration. Lower panel: Clinical status by Karnofsky scale compared to time following original surgery and in relationship to immunotherapy (■) and chemotherapy (█) treatments. (↓)=death.

The 3 patients who received satisfactory immunotherapy courses all underwent second operations at 10–25 months following the initial procedure (Table IV). In none of the surgical or autopsy examinations was there any evidence of an allergic encephalomyelitis despite specific attention to this possible development.

All 3 patients developed varying degrees of anaemia and leukopenia during their therapy. Patient A.F. required three transfusions in temporal association with chemotherapy. During this same time period and white count ranged from 13,100 to 1,800 with a progressive decline during the first 10 months of therapy stabilizing at between 5,000 and 2,200. Platelet counts during this time period ranged between 381,000 and 53,000, the degree of thrombocytopenia roughly correlating with the degree of leucopenia. On 5 occasions, the white count was measured at the time of immunization and 1 week subsequently. On 4 of those 5 occasions, a 5–44% decrease in total white count was noted 1 week following immunization. The second patient, A.J., maintained a haemoglobin between 10.4–14.4 during the first 12 months of therapy. During this time period, the white count was between 2,800 and 4,800 and the platelet count from 61,000 to 363,000. Twelve months following surgery, after receiving the 8th immunization, the patient's haemoglobin dropped from 11.3 to 5.8 over 5 days. The white count ranged from

**Table IV** Pathological changes

| *Patient* | *First Operation* | *Second Operation* | *Autopsy* |
|---|---|---|---|
| MP | Anaplastic Gemistocytic Astrocytoma | Astrocytoma | Residual Tumour Radiation Changes |
| AJ | Glioblastoma Multiforme | Glioblastoma Multiforme | Residual Glioma |
| AF | Glioblastoma Multiforme | Astrocytoma | Residual Tumour |

1,300 to 2,000 and the platelet count was from 50,000 to 79,000. At this time, the patient developed a generalized rash and the Westergren sedimentation rate, which had ranged from 11 to 33 rose to between 43 and 163. The patient was admitted to hospital for an evaluation of the anaemia and possible sepsis. Multiple cultures including sputum, blood, cerebrospinal fluid, urine and marrow were all negative. A sample of bone marrow demonstrated a generalized depression compatible with cytotoxic drugs. The patient's anaemia spontaneously cleared and the haemoglobin was stabilized at 11.1–11.6 with white counts from 2,600–3,400 and platelet counts from 61,000–130,000. The Westergren sedimentation rate continued, however, to be elevated at 92–108. The patient expired within one month with evidence of herniation. The third patient, M.P., maintained a haemoglobin of 11.7–15.2, a white count of 3,300–6,800, a platelet count of 85,000–290,000 and sedimentation rates of 6–20 during the entire course of therapy.

Of the 3 patients receiving immunotherapy, two, A.F. and M.P., produced levels of cytotoxic antibody against the immunizing cell line U251-MG (Figure 1). The 3rd patient (A.J.) produced only negligible levels of antibody as detected by [$^{14}$C]nicotinamide release assay throughout the course of the immunotherapy. As shown in Figure 1, the responses of patients A.F. and M.P. were quite similar, peaking between the 5th and 6th immunizations, followed by an abrupt and irreversible fall in detectable complement-dependent cytolytic antibody. In both cases, the observed drop in cytolytic activity followed the 4th course of chemotherapy.

The antibody titres of both patients at the peak of their respective responses were low (Figure 2). The 50% specific release endpoint of unabsorbed serum was 1/32 for patient A.F. and ~1/100 for patient M.P. Because of low antibody titre, serial absorption to completeness as performed by Mahaley *et al.* (1983) was not done; instead, either two absorptions with pooled human PBL ($2\times10^7$ cells) or a single absorption with $10^7$ 2-T sarcoma cells of peak response sera from patients A.F. and M.P. were performed. Either absorbent removed ≥50% of the cytolytic activity of both of these sera (Figure 2), indicating that although a significant cytolytic antibody response was detected,

**Figure 2** Antibody titres of patients (a) A.F. and (b) M.P. at the peak of their respective serologic responses in a [$^{14}$C]-nicotinamide release assay against immunizing cell line U-251 MG. The 50% specific release end-point of unabsorbed serum was 1/32 for patient A.F. and ~1/100 for patient M.P. (●) unabsorbed; (○) PBL-absorbed; (□) sarcoma 2T-absorbed the predominant cytolytic activity was not specific for the immunogen.

In our small series of patients, a high degree of selection was employed. This was done in order to minimize the potential for possible tumour growth of the viable immunizing cell line and to maximize the potential for response. Of the 3 patients that were serially immunized, the 2 younger patients demonstrated an early serologic response to the immunizing cell line. As demonstrated in previous studies immunizing non-human primates (Wikstrand & Bigner, 1977; Wikstrand *et al.*, 1979), the bulk of the antibody response to cultured glioma cells was directed against non-immunogen specific antigens such as; HLA-antigens, FCS-adsorbed specificities and other nonspecific antigens with a small, but detectable, amount of reactivity to immunogen remaining following absorption with non-glioma human cell absorbents; normal brain, PBL, and control non-glioma cultured tumour cells. This is in contrast to the results seen by Bloom *et al.* (1973) utilizing autologous irradiated glioma cells. However, in that study only 10/27 patients received multiple inoculations and no patients received adjuvant. In contrast, Trouillas & Lapras (1969) found identifiable immunodiffusion precipitation reactions with autologous tumour extracts in 14/20 patients whom they immunized serially with autologous tumour tissue mixed with Freund's complete adjuvant. In subsequent work from that same group, positive DHR reactions were seen in 23/24 patients to glioma cell lines following immunization (Febvre *et al.,* 1972). Whether the selection of potentially immunocompetent patients plays a role in this response to therapy remains more speculative than the use of adjuvant. Other than the serologic response of these 2 patients and the local reactions, there was no evidence that a significant immunologic reaction occurred. In none of the 3 patients was there any leukocytosis, development of increased reaction to glioma extracts on LIF assay or an increase in lymphocytic infiltration of their tumours at autopsy or second surgical procedure. But because all 3 patients underwent cytotoxic chemotherapy during this period of time, it is hard to clearly assess the interaction between these two phenomena.

It may be speculated that the prolonged survival seen in the 2 patients who mounted serologic responses is related to the immunotherapy. However, these patients were young and had high initial Karnofsky scores, both of which influence the natural history of the disease. A similar finding of prolonged survival has been reported from a separate study using irradiated cells from this same immunizing cell line in combination with BCG-cw, Levamisole and chemotherapy with BCNU (Mahaley *et al.,* 1983). In that study, 20 patients with malignant gliomas were selected for active immunization with either U251-MG cell line or D54-MG cell line, both of which were derived from malignant gliomas. Patients who were inoculated with the U251-MG cell line had a longer survival time when compared to those inoculated with D54-MG cell line or when compared with historical controls treated with Levamisole, radiation therapy and chemotherapy. Differences in titre between viable *versus* irradiated cells were not significantly different when both were used in non-human primates (Wikstrand & Bigner, 1981). This appears to also be true in humans, although only limited data exist on this point. Although no toxicity was seen in this study utilizing live cells, the lack of definite advantage and the potential for complications would suggest that future studies should display irradiated cells. While no definitive conclusions can be drawn from these 2 small series, further clinical and immunologic evaluation of active immunotherapy would appear to be warranted.

The question of haematologic compromise is also suggested from the data obtained in these patients. However, comparative haematologic data from 126 patients receiving similar chemotherapy were essentially identical. Patients undergoing immunotherapy were also subject to phlebotomy twice as often as chemotherapy patients, which may have played a significant role in their haematologic compromise. This, in association with the use of cytotoxic drugs, may explain this phenomenon. Why 2 of the 3 patients also developed episodes of anaemia in association with elevated sedimentation rates is unexplained despite extensive evaluation. No evidence of infection was seen and only generalized bone marrow depression compatible with chemotherapy was noted. At autopsy, neither of these patients demonstrated any evidence of allergic encephalomyelitis or a significant autoimmune reaction.

In summary, adjuvant therapy of malignant gliomas with viable, HLA mismatched glioma cells appears to be a safe form of therapy that deserves further evaluation because of its theoretical potential. In this study, two of three patients receiving immunotherapy had prolonged survival and evidence of a serologic response to the immunizing cell line. No evidence of local tumour growth or an allergic encephalitis was seen.

We would like to thank Prof. W.I. MacDonald, Prof. L. Symon, Mr N. Hoyle, Mr N. Shannon, Mr A. Crockard and the nursing staff of the National and Maida Vale Hospitals for their assistance and encouragement. Dr D. Bullard was the recipient of the National Research Service Award 1-31-CA 06680-01 from the National Cancer Institute. Drs J. Darling and D.G.T. Thomas are supported by the Cancer Research Campaign and the Brain Research Trust.

**References**

ALBERT, E. & MAYR, W. (eds). (1984). *Histocompatibility Testing,* Heidelberg: Springer Verlag (in press).

ALBRIGHT, L., SEAB, J.A. & OMMAYA, A.K. (1977). Intracerebral delayed hypersensitivity reactions in glioblastoma multiforme patients. *Cancer,* **39,** 1331.

BEAN, J.R., DARLING, J.L., HOYLE, N.R., ARIGBABU, S.O. & THOMAS, D.G.T. (1983). Alterations in the cellular immune response of patients with cerebral glioma, benign intracranial tumour, and spontaneous subarachnoid haemorrhage measured *in vitro* by the leucocyte migration inhibition test. *Neurol. Res.,* **5,** 61.

BIGNER, D.D., BIGNER, S.H., PONTEN, J. & 6 others. (1981*a*). Heterogeneity of genotypic and phenotypic characteristics of fifteen permanent cell lines derived from human gliomas. *J. Neuropathol. Exp. Neurol.*, **40**, 201.

BIGNER, D.D., PITTS, O.M. & WIKSTRAND, C.J. (1981*b*). Induction of lethal experimental allergic encephalomyelitis in non human primates and guinea pigs with human glioblastoma multiforme tissue. *J. Neurosurg.*, **55**, 32.

BLOOM, W.H., CARSTAIRS, K.C., CROMPTON, M.R. & McKISSOCK, W. (1960). Autologous glioma transplantation. *Lancet*, **ii**, 77.

BLOOM, H.J.G., PECKHAM, M.J., RICHARDSON, A.E., ALEXANDER, P.A. & PAYNE, P.M. (1973). Glioblastoma multiforme; a controlled trial to assess the value of specific active immunotherapy in patients treated by radical surgery and radiotherapy. *Br. J. Cancer*, **227**, 253.

BODMER, W.F., BATCHELOR, J.R., BODMER, J.G., FESTENSTEIN, H. & MORRIS, P.J. (eds). (1978). *Histocompatibility Testing*, 1977. Report of the 7th International Histocompatibility Testing Workshop Conference. Copenhagen: Munksgaard.

BODMER, W.F. & BODMER, J.G. (1979). Cytofluorochromasia for HLA-A, B, C and Dr typing. In: *NAIAD Manual of Tissue Typing Techniques*. (Ed. ????), NIH Publication 80–545, US Dept. of Health, Education and Welfare, p. 46.

BROOKS, W.H., MARKESBERY, W.R., GUPTA, G.D. & ROSZMAN, T.L. (1978). Relationship of lymphocyte invasion and survival of brain tumour patients. *Ann. Neurol.*, **4**, 219.

BROOKS, W.H., NETSKY, M.G., NORMANSELL, D.E. & HORRWITZ, D.A. (1972). Depressed cell-mediated immunity in patients with primary intracranial tumours. Characterization of a humoral immunosuppressive factor. *J. Exp. Med.*, **136**, 1631.

BROOKS, W.H., ROSZMAN, T.L., MAHALEY, M.S., Jr., & WOOSLEY, R.E. (1977). Immunobiology of primary intracranial tumours. II. Analysis of lymphocyte subpopulations in patients with primary brain tumours. *Clin. Exp. Immunol.*, **29**, 61.

BROOKS, W.H., ROSZMAN, T.L. & ROGERS, A.S. (1976). Impairment of rosette-forming T lymphocytes in patients with primary intracranial tumors. *Cancer*, **37**, (Part 2):1869.

BULLARD, D.E., BIGNER, S.H. & BIGNER, D.D. (1981*a*). The morphologic response of cell lines derived from human gliomas to dibutryl adenosine 3′:5′ cyclic monophosphate. *J. Neuropathol. Exp. Neurol.*, **40**, 230.

BULLARD, D.E., SCHOLD, S.C., Jr., BIGNER, S.H. & BIGNER, D.D. (1981*b*). Growth and chemotherapeutic response in athymic mice of tumors arising from human glioma-derived cell lines. *J. Neuropathol. Exp. Neurol.*, **40**, 410.

FEBVRE, H., MAUNOURY, R., CONSTANS, J.P. & TROUILLAS, P. (1972). Reactions d'hypersensibilite retardee avec des lignees de cellules tumorales humaines cultivees *in vitro* chez des malades porteurs de tumeurs cerebrales malignes. *Int. J. Cancer*, **10**, 221.

GRACE, J.T., Jr., PERESE, D.M., METZGAR, R.S., SASABE, T. & HOLDRIDGE, B. (1961). Tumor autograft responses in patients with glioblastoma multiforme. *J. Neurosurg.*, **18**, 159.

GREEN, S.B., BYAR, D.P., WALKER, M.D. & 15 others. (1983). Comparisons of carmustine, procarbazine, and high dose methylprednisolone as additions to surgery and radiotherapy for the treatment of malignant glioma. *Cancer Treat. Rep.*, **67** (Part 1), 121.

KARNOFSKY, D.A., ABELMANN, W.H., CRAVER, L.F. & BURCHENAL, J.H. (1948). The use of the nitrogen mustards in the palliative treatment of carcinoma: With particular reference to bronchogenic carcinoma. *Cancer*, **1**, 634.

LEVIN, V.A., CRAFTS, D.C., NORMAN, D.M., HOFFER, P.B., SPIRE, J.P. & WILSON, C.B. (1977). Criteria for evaluating patients undergoing chemotherapy for malignant brain tumors. *J. Neurosurg.*, **47**, 329.

MAHALEY, M.S., Jr., BIGNER, D.D., DUDKA, L.F. & 5 others. (1983). Immunobiology of primary intracranial tumors. Part 7: Active immunization of patients with anaplastic human glioma cells – a pilot study. *J. Neurosurg.*, **59** (Part 1), 201.

MAHALEY, M.S., Jr., BROOKS, W.H., ROSZMAN, T.L., BIGNER, D.D., DUDKA, L. & RICHARDSON, S. (1977). Immunobiology of primary intracranial tumors. Part 1: Studies of the cellular and humoral general immune competence of brain-tumor patients. *J. Neurosurg.*, **46**, 467.

MAHALEY, M.S., Jr., GILLESPIE, G.Y., GILLESPIE, R.P. & 5 others. (1983). Immunobiology of primary intracranial tumors. Part 8: Serological responses to active immunization of patients with anaplastic gliomas. *J. Neurosurg.*, **59** (Part 1), 208.

RUSSELL, D.C. & RUBINSTEIN, L.J. (1977). *Pathology of Tumours of the Nervous System*, Baltimore: William & Wilkins Co.

SCHOLD, S.C., Jr. (1981). Chemotherapy of primary central nervous system neoplasm. *Sem. Neurol.*, **1**, 189.

TERASAKI, P.I. (ed). (1980). *Histocompatibility Testing*, 1980. Report of the 8th International Histocompatibility Workshop, Los Angeles, USA. UCLA Tissue Typing Laboratory, Los Angeles, California.

TROUILLAS, P. (1973). Immunologie et immunotherapie des tumeurs cerebrales. Etat actuel. *Rev. Neurol.*, **128**, 23.

TROUILLAS, P. & LAPRAS, C. (1969). L'immunotherapie cellulaire des glioblastomes cerebraux. A propos de deux resultats. *Le Journal de Medecine de Lyon*, **1172**, 1269.

WIKSTRAND, C.J. & BIGNER, D.D. (1979). Surface antigens of human glioma cells shared with normal adult and fetal brain. *Cancer Res.*, **39**, 3235.

WIKSTRAND, C.J. & BIGNER, D.D. (1980). Immunobiological aspects of the brain and human gliomas; A review. *Am. J. Pathol.*, **98**, 515.

WIKSTRAND, C.J. & BIGNER, D.D. (1981). Hyper-immunization of nonhuman primates with BCG-CW and cultured human glioma-derived cells: Production of reactive antisera and absence of EAE induction. *J. Neuroimmunol.*, **1**, 249.

WIKSTRAND, C.J., MAHALEY, M.S., Jr., & BIGNER, D.D. (1977). Surface antigenic characteristics of human glial brain tumour cells. *Cancer Res.*, **37**, 4267.

YOUNG, H.F., KAPLAN, A. & REGELSON, W. (1977). Immunotherapy with autologous white cell infusions ("lymphocytes") in the treatment of recurrent glioblastoma multiforme. A preliminary report. *Cancer*, **40**, 1037.